# Early wounds, minds in training: childhood adversity and the mediating effect of psychological distress in suicidal risk among medical students

**DOI:** 10.3389/fpsyg.2026.1970728

**Published:** 2026-10-21

**Authors:** Gabriela A. Martínez-Levy, Ana Fresán-Orellana, Magdalena Briones-Velasco, Pedro G. Romero-Silva, Diana P. Guizar-Sánchez

**Affiliations:** 1 Departamento de Genética, Dirección y Subdirección de Investigaciones Biomédicas en Salud Mental, Instituto Nacional de Psiquiatría Ramón de la Fuente Muñiz, Ciudad de México, Mexico; 2 Laboratorio de Epidemiología Clínica, Dirección y Subdirección de Investigaciones Biomédicas en Salud Mental, Instituto Nacional de Psiquiatría Ramón de la Fuente Muñiz, Ciudad de México, Mexico; 3 Departamento de Fisiología, Facultad de Medicina, Universidad Nacional Autónoma de México, Ciudad de México, Mexico

**Keywords:** academic stress, anxiety, childhood adversity, childhood maltreatment, depression, mediation analysis, medical students, suicide risk

## Abstract

**Background:**

Childhood adversity is a well-established distal determinant of suicide risk, yet the proximal psychological mechanisms linking it to suicidal vulnerability during medical training remain poorly understood.

**Objective:**

To examine whether depressive symptoms, anxiety symptoms, and perceived academic stress mediate the association between childhood emotional and physical maltreatment with suicide risk among medical students.

**Methods:**

This cross-sectional study included 670 second year medical students from a large public university in Mexico, assessed with validated measures of childhood maltreatment (CTQ-SF), depressive symptoms (PHQ-9), anxiety symptoms (GAD-7), academic stress (SISCO Inventory), and suicide risk (Plutchik Suicide Risk Scale). Group comparisons used chi-square and independent-samples *t* tests; parallel multiple mediation analyses were conducted using PROCESS (Model 4) with 5,000 bias-corrected bootstrap resamples.

**Results:**

Overall, 26.0% of students were classified at high suicide risk (Plutchik score ≥ 6). Compared with low-risk students, those at high risk reported greater childhood emotional and physical maltreatment, more severe depressive and anxiety symptoms, and higher academic stress. Parallel mediation analyses showed significant indirect effects of both emotional and physical maltreatment on suicide risk through psychological distress, with bootstrap 95% confidence intervals excluding zero. All three specific indirect pathways through depressive symptoms, anxiety symptoms, and academic stress were statistically significant in both models, with depressive symptoms showing the largest specific indirect effects [emotional: *B* = 0.08, 95% CI (0.06, 0.10); physical: *B* = 0.12, 95% CI (0.08, 0.16)].

**Conclusions:**

Depressive symptoms, anxiety symptoms, and academic stress partially mediate the association between childhood maltreatment and suicide risk among medical students. These findings identify psychological distress as a clinically relevant and potentially modifiable pathway linking early adversity to suicide risk and support trauma-informed prevention strategies that integrate early detection and management of psychological distress within medical education.

## 1 Introduction

Medical training is characterized by sustained academic pressure, sleep deprivation, perfectionistic expectations, and emotionally demanding learning environments (Eley and Slavin, 2024), conditions that may contribute to the substantial mental health burden observed among medical students. Medical students experience higher rates of depression, anxiety, burnout, sleep disturbances, and suicidal behaviors than their age-matched peers, making suicide prevention a growing priority in medical education (Carrard et al., 2024; Peng et al., 2023; Rotenstein et al., 2016). Meta-analytic evidence estimates the prevalence of depression among medical students at 27.2%−28.0% and suicidal ideation at 11.1%, although substantial variability exists across training stages, geographic regions, and study methodologies (Puthran et al., 2016; Rotenstein et al., 2016). Recent evidence further demonstrates considerable heterogeneity in suicidal outcomes across medical student populations, with reported lifetime suicidal ideation ranging from 2.9% to 53.6% and suicide attempts from 1.6% to 8% (Agyapong-Opoku et al., 2025; Coentre and Góis, 2018; Mateen et al., 2024; Song et al., 2026). This variability suggests that exposure to the demands of medical training alone may not fully explain differences in vulnerability to psychological distress and suicide risk.

Indeed, students exposed to similar educational demands may experience markedly different psychological outcomes, suggesting that current stressors may interact with vulnerabilities established before medical school. Consistent with this perspective, a meta-analysis of risk factors for suicidal ideation and suicide attempts among medical students identified depression, burnout, comorbid mental illness, and stress among the strongest correlates of suicidality (Seo et al., 2021). These findings highlight the importance of considering both pre-existing developmental vulnerability and proximal, potentially modifiable psychological factors when examining suicide risk during medical training.

Developmental models of suicide propose that suicidal behavior arises from the interaction between distal vulnerabilities and proximal psychological mechanisms, rather than from exposure to a single precipitating factor (Turecki et al., 2019; Turecki and Brent, 2016). Childhood maltreatment represents an important distal vulnerability because adverse experiences during sensitive developmental periods may have persistent effects on emotional regulation, cognitive development, and stress responsivity across the lifespan (Du et al., 2026; McLaughlin et al., 2010; Teicher et al., 2016). Meta-analytic and longitudinal evidence consistently link childhood maltreatment with later depression, anxiety, suicidal ideation, and suicide attempts. Recent causal-inference evidence further suggests that childhood maltreatment is strongly associated with major depressive disorder, anxiety, post-traumatic stress disorder, and non-fatal self-harm, while its direct association with suicide attempts may be less pronounced (Zhang et al., 2025). Consistent with this framework, Yao et al. (2023) found that mental distress, characterized by depressive and anxiety symptoms, played a mediating role in the association between childhood trauma and suicide risk among young adults. Together, these findings support the possibility that the association between childhood maltreatment and suicide risk may operate, at least partly, through proximal psychological processes.

Evidence from medical student populations supports this developmental perspective. In a longitudinal study of 2,192 medical students, emotional neglect, physical neglect, emotional abuse, and physical abuse predicted persistently elevated trajectories of suicidal ideation over time (Nan et al., 2023). More recently, childhood trauma has been associated with suicidal ideation among medical students through psychological pathways involving alexithymia and resilience, suggesting that the relationship between early adversity and suicidality may be partly explained by modifiable psychological processes (Gao et al., 2025). Most of the empirical evidence examining these pathways, however, has been generated in East Asian samples, particularly among Chinese medical students, where alexithymia, resilience, and emotion dysregulation have emerged as relevant mediators (Gao et al., 2025; Nan et al., 2023). Complementary evidence from Western health professions education has shown that adverse childhood experiences are associated with greater psychological distress and has examined resilience as a potential mechanism underlying this relationship, although evidence for a mediating role of resilience remains inconclusive (Sciolla et al., 2025).

This relationship is particularly relevant in Latin America, where childhood adversity and mental health problems represent substantial public health concerns. In Mexico, 88.2% of adults reported at least one adverse childhood experience and 22.6% reported four or more, with greater cumulative exposure associated with markedly higher odds of depression and anxiety (León Rojas et al., 2025; Sánchez-Jáuregui et al., 2023). Among Latin American medical students, meta-analytic evidence has estimated the prevalence of suicidal ideation at approximately 13.8%, further underscoring the mental health burden within this population (Denis-Rodríguez et al., 2017). Despite this convergence of substantial childhood adversity and elevated suicide risk, the psychological mechanisms connecting these phenomena during medical training remain insufficiently characterized in Latin America. To our knowledge, no study has specifically modeled the pathways through which childhood maltreatment may contribute to suicide risk among Latin American medical students. Addressing this context-specific gap is therefore important for identifying potentially modifiable mechanisms and informing prevention strategies tailored to medical education settings in the region.

Depressive symptoms, anxiety symptoms, and academic stress are especially relevant candidate mediators because they are prevalent during medical training, closely associated with suicide risk, and potentially amenable to intervention. Depression is among the strongest proximal correlates of suicidal ideation and suicide attempts in medical students (Seo et al., 2021), while anxiety has also been shown to mediate associations between childhood maltreatment and suicidal outcomes independently of depressive symptoms (Chen et al., 2021). Importantly, these psychological processes are also closely interconnected and may mutually reinforce one another. Academic stress may contribute to depressive and anxiety symptoms, whereas these symptoms may, in turn, heighten the perception of academic demands and undermine coping resources, potentially sustaining a cycle of distress that increases suicide risk. For example, chronic academic stress may contribute to depressive and anxiety symptoms, heightened anxiety may amplify the perception of academic demands as stressful, and depressive symptoms may erode the coping resources needed to manage those demands, potentially sustaining a cycle of psychological distress. Academic stress may represent a particularly relevant contextual mechanism during medical training. Early adversity may heighten sensitivity to subsequent stressors (McLaughlin et al., 2010), and previous mediation research in university students has identified perceived stress as a proximal pathway linking childhood trauma with suicide-related outcomes (Wu et al., 2022). Within medical education, where sustained academic demands represent a major source of stress, academic stress may constitute a context-specific manifestation of this broader stress pathway and has been associated with suicidal ideation and psychological distress (Pourmohammad et al., 2026; Tyagi et al., 2026). Examining depressive symptoms, anxiety symptoms, and academic stress simultaneously may therefore provide a more comprehensive account of how distal developmental vulnerability is associated with current suicide risk. Therefore, the aim of the present study is to examine whether depressive symptoms, anxiety symptoms, and perceived academic stress mediate the association between childhood emotional and physical maltreatment with suicide risk among medical students in Mexico.

## 2 Materials and methods

### 2.1 Study design

This cross-sectional study was conducted between February and March 2025 among second-year medical students at the Faculty of Medicine, National Autonomous University of Mexico (UNAM). The study was designed and reported in accordance with the Strengthening the Reporting of Observational Studies in Epidemiology (STROBE) guidelines.

### 2.2 Participants and procedures

The source population comprised approximately 2,215 second-year medical students enrolled at the Faculty of Medicine, UNAM, during the 2025 academic year. Second-year students were selected because this stage of training is characterized by increased academic demands and represents a critical period for the emergence of psychological distress.

Participants were recruited during regularly scheduled classroom sessions. Eligible individuals were required to be 18 years or older and provide voluntary consent to participate. Questionnaires with 10% or more missing data were excluded from the final analysis. Of the 682 students who agreed to participate, 670 provided complete data and were included in the final analysis, representing approximately 30% of the eligible population. After providing written informed consent, participants completed a battery of self-administered questionnaires under the supervision of trained research staff. The assessment required approximately 30–40 min.

### 2.3 Measures and operational definitions

The study assessed childhood maltreatment as the primary exposure, depressive symptoms, anxiety symptoms and perceived academic stress as parallel mediators, and suicide risk as the outcome. All constructs were assessed using validated self-report instruments.

### 2.4 Childhood maltreatment

Childhood maltreatment was assessed using the Childhood Trauma Questionnaire–Short Form (CTQ-SF), a 28-item retrospective self-report instrument assessing experiences of abuse and neglect before 18 years of age across five domains: emotional abuse, physical abuse, sexual abuse, emotional neglect, and physical neglect. Items are rated on a five-point frequency Likert scale, with higher scores indicating greater severity of childhood maltreatment (Bernstein et al., 2003). Consistent with established CTQ-SF scoring criteria, each subscale was classified into four severity categories: none, mild, moderate and severe. The thresholds for the moderate-to-severe category were ≥13 for emotional abuse, ≥10 for physical abuse, ≥8 for sexual abuse, ≥15 for emotional neglect, and ≥10 for physical neglect (Bernstein and Fink, 1998).

For the primary mediation analyses, two continuous composite variables were specified *a priori*. Emotional maltreatment was calculated as the sum of the emotional abuse and emotional neglect subscale scores, whereas physical maltreatment was calculated as the sum of the physical abuse and physical neglect subscale scores. These continuous composite scores were used as the exposure variables in two separate parallel mediation models. Sexual abuse was not included in either composite because it represents a conceptually distinct form of maltreatment; however, it was retained as an individual CTQ-SF domain in descriptive analyses.

For descriptive comparisons, each CTQ-SF subscale was additionally dichotomized as none/mild (none/minimal and low/moderate) or moderate/severe (moderate/severe and severe/extreme). The CTQ-SF has demonstrated adequate-to-excellent psychometric properties in Spanish-speaking populations (Cronbach's α = 0.66–0.94) (Hernandez et al., 2013; Martín-Babarro et al., 2021).

### 2.5 Depressive symptoms

Depressive symptoms were assessed using the Patient Health Questionnaire-9 (PHQ-9), a nine-item self-report instrument evaluating depressive symptoms experienced during the previous two weeks. Items are scored from 0 to 3, yielding a total score ranging from 0 to 27, with higher scores indicating greater depressive symptom severity (Kroenke et al., 2001).

A cutoff score of ≥7 was used to identify clinically relevant depressive symptoms, consistent with validation studies in Latin American populations. The PHQ-9 has demonstrated good reliability in Spanish-speaking and Mexican populations (Cronbach's α ≈ 0.78–0.90) (Arrieta et al., 2017; Díaz De León Castañeda and Díaz-Castro, 2025). For mediation analyses, the PHQ-9 total score was analyzed as a continuous variable.

### 2.6 Anxiety symptoms

Anxiety symptoms were assessed using the Generalized Anxiety Disorder-7 (GAD-7), a seven-item self-report instrument evaluating anxiety symptoms during the previous two weeks. Items are scored from 0 to 3, yielding a total score ranging from 0 to 21, with higher scores indicating greater symptom severity (Spitzer et al., 2006). A cutoff score of ≥10 was used to identify clinically significant anxiety symptoms, consistent with the original validation study. The GAD-7 has demonstrated excellent reliability in Mexican populations (Cronbach's α ≈ 0.88–0.94), including nationally representative samples, culturally adapted versions for clinical populations, and Mexican migrant samples (Galindo-Vázquez et al., 2026; Garcia-Campayo et al., 2010; Gutiérrez-Velilla et al., 2022). The continuous GAD-7 total score was used in the mediation analyses.

### 2.7 Perceived academic stress

Perceived academic stress was assessed using the SISCO Academic Stress Inventory, a validated self-report instrument designed for university students. The instrument assesses exposure to academic stressors and associated psychological, physical, and behavioral responses to academic demands, with higher scores indicating greater perceived academic stress. The SISCO Inventory has demonstrated good internal consistency in Latin American university populations (Cronbach's α = 0.82–0.86) (Barraza Macías, 2007; Guzmán-Castillo et al., 2022). The total SISCO score was analyzed as a continuous variable in the mediation models.

### 2.8 Suicide risk

Suicide risk was assessed using the Plutchik Suicide Risk Scale, a 15-item self-report instrument evaluating suicidal ideation, previous suicide attempts, hopelessness, and other suicide-related risk factors. Total scores range from 0 to 15, with higher scores indicating greater suicide risk.

Consistent with validation studies in Mexican populations, a cutoff score of ≥6 was used to classify participants as having high suicide risk for descriptive and group-comparison analyses. The scale has demonstrated satisfactory internal consistency (Cronbach's α ≈ 0.74–0.80) (Santana-Campas and Santoyo Telles, 2018; Tapia Varela et al., 2025). For mediation analyses, the Plutchik total score was treated as a continuous outcome.

In the present sample, internal consistency was: CTQ-SF total α = 0.90 (emotional abuse α = 0.82; emotional neglect α = 0.86; physical abuse α = 0.81; physical neglect α = 0.79; sexual abuse α = 0.90); PHQ-9 α = 0.87; GAD-7 α = 0.90; SISCO α =0.80; Plutchik Suicide Risk Scale α = 0.78, supporting adequate psychometric performance of all instruments within this specific cohort.

### 2.9 Ethical considerations

The study protocol was approved by the Research and Ethics Committee of the Faculty of Medicine, National Autonomous University of Mexico (Approval No. FM/DI/089/2024). All procedures were conducted in accordance with the ethical principles of the Declaration of Helsinki and its subsequent amendments. Prior to enrollment, participants received written and verbal information regarding the study objectives, procedures, voluntary nature of participation, and confidentiality of the collected data, and all provided written informed consent. Data was de-identified before statistical analyses to ensure participants' privacy and confidentiality. Given the sensitive nature of the assessment, students who reported psychological distress, elevated suicide risk, or requested assistance were informed about the university's available mental health resources and referred, when appropriate, to institutional psychological or psychiatric services according to established referral procedures.

### 2.10 Statistical analysis

The description of demographic characteristics, psychological distress and childhood adversity variables was conducted using frequencies and percentages for categorical variables and means and standard deviations (SD) for continuous variables. Chi–square tests and independent-sample Student's *t*-tests were used to compare these variables between suicide risk groups (low risk vs. high risk). Effect sizes for the comparative analyses were calculated using Cramer's V for chi–square tests and Cohen's d for *t*-tests, with interpretations as small (0.1–0.3), medium (0.4–0.7), and large (>0.8).

Following Hayes' procedure, the PROCESS macro for SPSS (version 4.2; https://afhayes.com/introduction-to-mediation-moderation-and-conditional-process-analysis.html) was employed to test the mediation hypotheses. Two parallel multiple mediation models (Model 4) were tested separately for emotional and physical maltreatment. In each model, the corresponding maltreatment measure was specified as the predictor, suicide risk as the outcome, and academic stress, depressive symptoms, and anxiety symptoms as simultaneous mediators.

For each model, the associations between maltreatment and each mediator (a paths), the associations between each mediator and suicide risk while controlling for the predictor and the other mediators (b paths), the total effect of maltreatment on suicide risk, and the direct effect after accounting for the mediators were estimated. The proportion of variance explained (*R*^2^) was obtained for each mediator model and for the final suicide risk model. Specific indirect effects through academic stress, depressive symptoms, and anxiety symptoms, as well as the total indirect effect, were estimated using 5,000 bootstrap samples. An indirect effect was considered statistically significant when its 95% percentile bootstrap confidence interval did not include zero.

Standardized regression coefficients (β) were used to represent the a and b paths and the total and direct effects in the mediation figures. Unstandardized indirect effects and their corresponding 95% percentile bootstrap confidence intervals were reported in the accompanying tables. The persistence of a statistically significant direct effect in the presence of a significant total indirect effect was interpreted as evidence of partial mediation.

The alpha level was set at *p* < 0.05. All statistical analyses were conducted using SPSS version 21 for PC.

## 3 Results

### 3.1 Demographics, childhood adversity and psychological distress between medical students with low and high suicide risk

The sample consisted of 670 students from the second year of medicine. The average age was 20.0 years (SD = 1.0; range = 18–27 years), with a higher proportion of students being women (69.4%; *n* = 465) and the vast majority, single (97.9%, *n* = 656).

The average total score of the Plutchik Suicide Risk scale was 4.4 (SD = 3.0, range 0–14). According to the cut-off points of the scale, 74.0% (*n* = 496) had a low suicide risk and the remaining 26.0% (*n* = 174), a high suicide risk.

Table 1 presents the descriptive characteristics of the total sample and the comparisons between students with low and high suicide risk. Compared with the low-risk group, participants in the high-risk group exhibited more severe symptoms of depression and anxiety, with a greater proportion of students reporting moderate to severe levels. Nearly all students in the high-risk group reported academic stress, and at higher intensity levels. Additionally, experiences of emotional and physical maltreatment were more prevalent in the high-risk suicide group. Sex, age and marital status were similar between groups. Age was not further stratified in group comparisons given the narrow and homogeneous age range of the sample (mean 20.0 years, SD 1.0–1.3); a supplementary comparison by age tercile is available on request. Sexual abuse, the CTQ-SF subdomain excluded from the mediation models, is reported descriptively in Table 1: mean (SD) sexual abuse scores were 5.7 (2.1) in the low-risk group and 7.7 (4.6) in the high-risk group. Using established CTQ-SF severity cutoffs, the proportion of participants classified as moderate/severe was higher in the high-risk group (28.2% vs. 9.1%; χ2 = 38.4, *p* < 0.001; Cramer's *V* = 0.2).

**Table 1:** Demographics, childhood adversity and psychological distress between medical students with low and high suicide risk.

Characteristic	Total *n* = 670	Low suicide risk *n* = 496	High suicide risk *n* = 174	Statistic
	*n* (%) / mean (SD)	*n* (%) / mean (SD)	*n* (%) / mean (SD)	
Demographic characteristics				
Sex—women	465 69.4	341 68.8	124 71.3	χ2 = 0.3, *p* = 0.53
Age^+^ (mean, SD)	20.0 1.3	20.0 1.0	20.0 1.9	*t* = −0.1, *p* = 0.90
Marital status–single	656 97.9	487 98.2	169 97.1	χ2 =0.7, *p* = 0.40
Psychological distress				
PHQ total score (mean, SD)	9.6 5.8	7.7 4.6	15.2 5.2	*t* = −17.7, *p* < 0.001
				Cohen's *d* = 1.5
PHQ groups				
No symptoms/mild	359 53.6	332 66.9	27 15.5	χ2 = 136.9, *p* < 0.001
Moderate/severe	311 46.4	164 33.1	147 84.5	Cramer's *V* = 0.4
GAD total score (mean, SD)	7.7 5.1	6.3 4.5	11.6 4.7	*t* = −13.0, *p* < 0.001
				Cohen's *d* = 1.1
GADgroups				
No symptoms/mild	444 66.3	384 77.4	60 34.5	χ2 = 106.2, *p* < 0.001
Moderate/severe	226 33.7	112 22.6	114 65.5	Cramer's *V* = 0.4
Inventory SISCO (n = 667)				
Academic stress – Yes	610 91.0	442 89.7	168 96.6	χ2 = 7.8, *p* = 0.005
				Cramer's *V* = 0.1
Stress intensity (mean, SD)	3.1 1.3	2.9 1.3	3.7 1.0	*t* = −7.2, *p* < 0.001
				Cohen's *d* = 0.6
Childhood adversity (mean, SD)				
Emotional maltreat total score	18.6 7.6	16.6 6.2	24.3 8.3	*t* = −12.7, *p* < 0.001
				Cohen's *d* = 1.0
Emotional maltreat groups^*^				
None/mild	491 73.3	416 83.9	75 43.1	χ2 = 109.3, *p* < 0.001
Moderate/severe	179 26.7	80 16.1	99 56.9	Cramer's *V* = 0.4
Emotional abuse score	9.3 4.3	8.2 3.4	12.6 4.8	*t* = −13.0, *p* < 0.001
				Cohen's *d* = 1.0
Emotional abuse groups^*^				
None/mild	525 78.4	437 88.1	88 50.6	χ2 = 106.8, *p* < 0.001
Moderate/severe	145 21.6	59 11.9	86 49.4	Cramer's *V* = 0.4
Emotional neglect score	9.2 4.2	8.4 3.7	11.6 4.5	*t* = −9.2, *p* < 0.001
				Cohen's *d* = 0.7
Emotional neglect groups^*^				
None/mild	581 86.7	456 91.9	125 71.8	χ2 = 45.1, *p* < 0.001
Moderate/severe	89 13.3	40 8.1	49 28.2	Cramer's *V* = 0.2
Physical maltreat total score	13.4 4.0	12.7 3.3	15.5 5.0	*t* = −8.2, *p* < 0.001
				Cohen's *d* = 0.7
Physical maltreat groups^*^				
None/mild	526 78.5	425 85.7	101 58.0	χ2 = 58.3, *p* < 0.001
Moderate/severe	144 21.5	71 14.3	73 42.0	Cramer's *V* = 0.3
Physical abuse score	6.8 2.8	6.4 2.4	7.9 3.5	*t* = −6.0, *p* < 0.001
				Cohen's *d* = 0.5
Physical abuse groups^*^				
None/mild	580 86.6	451 90.9	129 74.1	χ2 = 31.2, *p* < 0.001
Moderate/severe	90 13.4	45 9.1	45 25.9	Cramer's *V* = 0.2
Physical neglect score	6.6 2.1	6.3 1.8	7.6 2.6	*t* = −7.4, *p* < 0.001
				Cohen's *d* = 0.5
Physical neglect groups^*^				
None/mild	593 88.5	462 93.1	131 75.3	χ2 = 40.3, *p* < 0.001
Moderate/severe	77 11.5	34 6.9	43 24.7	Cramer's *V* = 0.2
Sexual abuse score (mean, SD)	6.2 (3.1)	5.7 (2.1)	7.7 (4.6)	*t* = −5.6, *p* < 0.001; Cohen's *d* = 0.7
Sexual abuse—none/mild	576 86	451 90.9	125 71.8	χ2 = 38.4, *p* < 0.001; Cramer's *V* = 0.2
Sexual abuse—moderate/severe	94 14	45 9.1	49 28.2	

^+^Data presented in years.

^*^Data presented in frequencies and percentages.

### 3.2 Mediation analysis for suicide risk in medical students

Since no significant differences were observed between groups in terms of demographic characteristics, the total scores from the PHQ (depressive symptoms), GAD (anxiety symptoms), and the SISCO Stress Inventory (academic stress) were included as simultaneous mediators of the association between maltreatment and suicide risk.

Figures 1, 2 present the mediation models for emotional and physical maltreatment, respectively. Both models showed significant total indirect effects on suicide risk through the proposed mediators. For emotional maltreatment, the model including emotional maltreatment and the three mediators explained 53.6% of the variance in suicide risk, whereas the corresponding physical maltreatment model explained 50.1%. All three specific indirect effects were statistically significant in both models. For emotional maltreatment, the indirect effects through academic stress, depressive symptoms, and anxiety symptoms were *B* = 0.005, *B* = 0.08, and *B* = 0.01, respectively; for physical maltreatment, the corresponding effects were *B* = 0.005, *B* = 0.12, and *B* = 0.01, with all 95% bootstrap confidence intervals excluding zero. Depressive symptoms accounted for the largest specific indirect effect in both models [emotional: *B* = 0.08, 95% CI (0.06, 0.10); physical: *B* = 0.12, 95% CI (0.08, 0.16)]. The total effects of emotional (β = 0.51, *p* < 0.001) and physical maltreatment (β = 0.33, *p* < 0.001) on suicide risk were significant, and the direct effects remained significant after inclusion of depressive symptoms, anxiety symptoms, and academic stress (emotional: β = 0.26, *p* < 0.001; physical: β = 0.14, *p* < 0.001), supporting partial mediation. (Total effects are estimated without the mediators; the values after their inclusion are the direct effects shown in Figures 1, 2). These findings indicate that students reporting higher levels of childhood adversity are more likely to exhibit elevated suicide risk, and that psychological distress contributes to suicide risk independently of childhood maltreatment.

**Figure 1:**
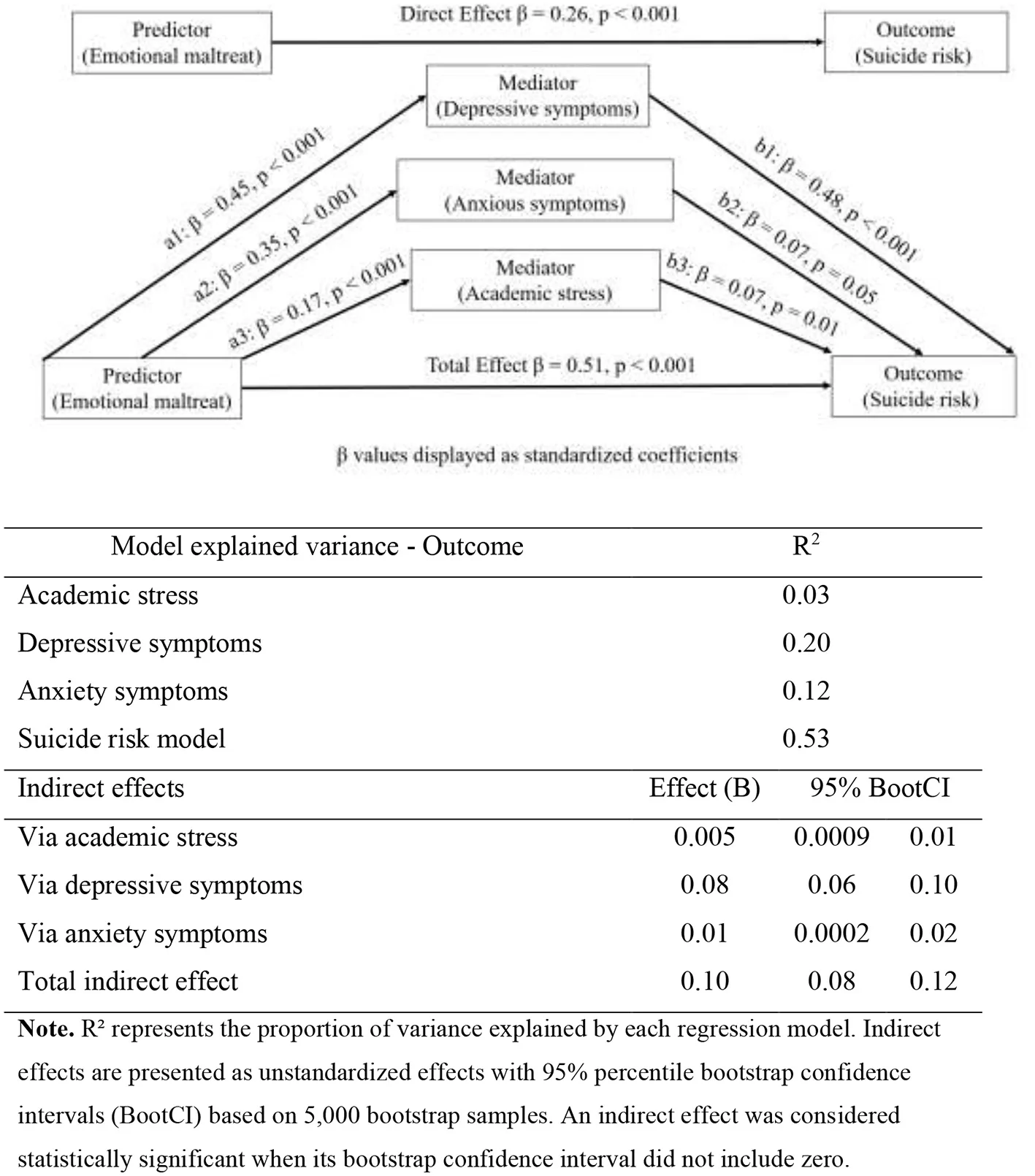
Results of the mediation model test of suicide risk by emotional maltreatment through the macro PROCESS.

**Figure 2:**
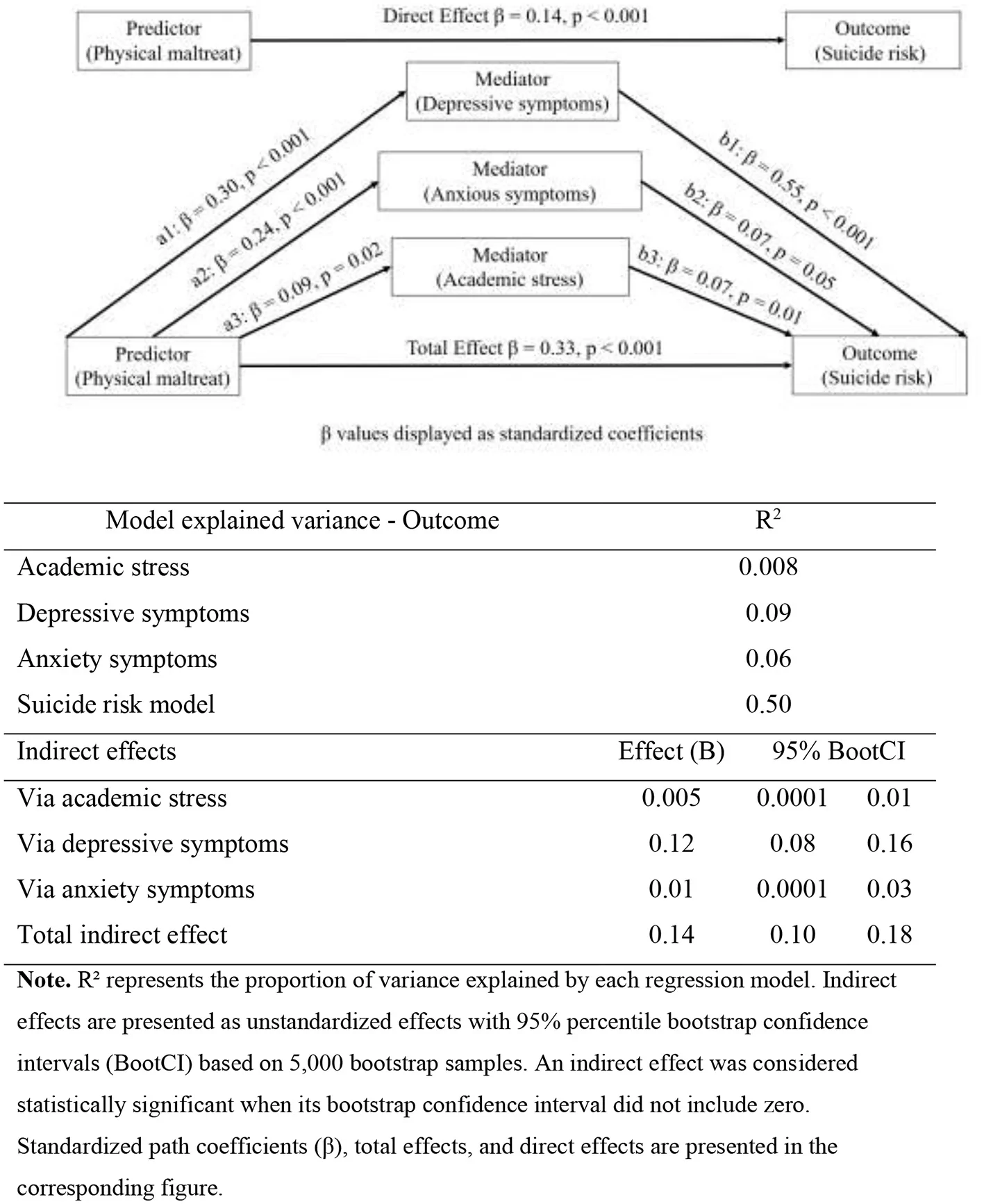
Results of the mediation model test of suicide risk by physical maltreatment through the macro PROCESS.

## 4 Discussion

The present study extends the growing body of evidence linking childhood adversity with suicidal behavior by demonstrating that the association between childhood emotional and physical maltreatment and suicide risk among medical students is partially explained by current psychological distress (depression, anxiety and academic stress). Mediation analyses indicated that these dimensions of psychological distress partially accounted for the association between childhood maltreatment and suicide risk, with depressive symptoms consistently emerging as the strongest mediating pathway for both emotional and physical maltreatment. These findings support contemporary developmental models proposing that early adverse experiences do not directly determine suicidal behavior but rather increase vulnerability through psychological processes that remain active during emerging adulthood (Turecki et al., 2019; Turecki and Brent, 2016).

Our findings are consistent with a large body of epidemiological evidence identifying childhood maltreatment as one of the most robust distal predictors of suicidal behavior. Meta-analytic studies have consistently shown that individuals exposed to childhood adversity exhibit approximately two- to threefold greater odds of suicidal ideation and suicide attempts than those without such experiences, even after adjustment for psychiatric disorders and other relevant clinical variables (Angelakis et al., 2020; Baldwin et al., 2023). This pattern is echoed specifically within medical student populations: a meta-analysis of suicide risk factors in this group identified stress, depression, burnout, and comorbid mental illness as the strongest correlates of suicidal ideation and attempt, all of which are consistent with the proximal, modifiable mediators examined here rather than the more distal exposure of childhood maltreatment itself (Seo et al., 2021). Rather than representing isolated traumatic events, adverse childhood experiences appear to alter developmental trajectories, increasing vulnerability to affective dysregulation, maladaptive cognitive schemas, and impaired stress responsiveness that may remain latent until challenged by subsequent environmental stressors (McLaughlin et al., 2010; Teicher et al., 2016).

Interestingly, we observed that both forms of childhood adversity were associated with suicide risk through psychological distress, but the relative magnitude of this indirect pathway differed depending on the metric considered: the standardized total effect was larger for emotional maltreatment (β = 0.51) than for physical maltreatment (β = 0.33), whereas the unstandardized total indirect effect was somewhat larger for physical maltreatment [*B* = 0.14, 95% CI (0.10, 0.18)] than for emotional maltreatment [*B* = 0.10, 95% CI (0.08, 0.12)]. Because the present study did not formally test the statistical difference between the two parallel mediation models, and because standardized and unstandardized comparisons yielded divergent patterns, we refrain from concluding that either maltreatment subtype has a definitively stronger indirect effect; this comparison should be interpreted with caution as an empirical observation rather than a confirmed differential effect. What was consistent across both models, however, is that depressive symptoms accounted for the largest specific indirect effect [emotional: *B* = 0.08, 95% CI (0.06, 0.10); physical: *B* = 0.12, 95% CI (0.08, 0.16)], underscoring depression as the principal proximal mechanism linking childhood maltreatment (regardless of subtype) to suicide risk. Notwithstanding this measurement-dependent pattern in the overall indirect effect, the direction observed for the standardized total effect is consistent with recent evidence suggesting that emotional abuse and emotional neglect may exert particularly pervasive effects on long-term emotional functioning. Unlike physical abuse, which primarily threatens bodily integrity, emotional maltreatment repeatedly undermines self-worth through rejection, humiliation, criticism, emotional invalidation, and the absence of supportive caregiving; because it targets the individual's developing identity rather than physical safety, it may be particularly likely to foster hopelessness, guilt, self-devaluation, and persistent negative self-schemas and self-criticism, characteristics repeatedly identified as central mechanisms linking childhood adversity with depression and suicidal ideation (Nelson et al., 2017; Ribeiro et al., 2018). Convergent evidence from young-adult samples supports this distinction: emotional abuse and emotional neglect have been shown to operate through mentalization and depression as mediators of suicide risk, whereas sexual abuse retains a significant direct effect independent of these psychological mechanisms, suggesting that different maltreatment subtypes may follow at least partly distinct pathways to suicidal vulnerability (Huang and Hou, 2023).

The prominent and consistent role of depressive symptoms as a mediator, together with the persistent contribution of emotional maltreatment, may be particularly relevant within medical education. Medical training places students in an environment characterized by constant evaluation, high expectations of achievement, perfectionistic standards, and repeated exposure to situations involving uncertainty and emotional burden. Individuals with histories of emotional maltreatment may be especially vulnerable to these contextual demands because academic criticism, perceived failure, or interpersonal conflict can reactivate maladaptive cognitive and emotional patterns established during childhood. Consequently, the educational environment may not create vulnerability *per se* but rather amplify developmental vulnerabilities acquired much earlier in life, consistent with developmental psychopathology frameworks proposing that early-life adversity interacts with later environmental stressors to increase susceptibility to psychopathology (Huang and Hou, 2023; McLaughlin et al., 2010; Teicher et al., 2016), and with systematic reviews of medical student suicide mortality identifying academic stress and perceived failure as the most frequently documented proximal motive, present in nearly 60% of reviewed suicide cases (Varshney et al., 2024).

Another noteworthy finding is that the direct effects of depressive symptoms, anxiety symptoms, and academic stress on suicide risk were stronger than the direct effects of childhood maltreatment itself. This observation reinforces the distinction between distal and proximal risk factors proposed by contemporary suicide theories (Turecki and Brent, 2016) and is compatible with causal-inference evidence indicating that childhood maltreatment predicts suicide attempts largely through its causal impact on depression and anxiety rather than through a direct causal pathway to suicidality (Zhang et al., 2025). From a clinical perspective, this distinction is particularly important because childhood maltreatment cannot be modified retrospectively, and this is arguably even more consequential precisely because the maltreatment itself is fixed; depressive symptoms, anxiety, and academic stress, by contrast, are dynamic and potentially treatable conditions, so identifying these proximal manifestations may provide valuable opportunities for early intervention before suicidal ideation progresses to more severe suicidal behaviors.

Collectively, these findings support a developmental interpretation of suicide risk among medical students. Rather than viewing suicidal behavior solely as the consequence of current academic stress or exclusively as the result of childhood adversity, the present study suggests that suicide risk emerges from the interaction between developmental vulnerability and contemporary psychological functioning. Medical school may therefore represent a critical developmental period during which latent vulnerabilities associated with childhood maltreatment become clinically expressed through depression, anxiety, and academic stress. Understanding this interaction provides a more comprehensive framework for suicide prevention than considering either developmental history or current psychological symptoms in isolation and underscores the importance of integrating trauma-informed approaches into mental health strategies within medical education.

### 4.1 Psychological distress as the proximal mechanism linking childhood maltreatment and suicide risk

Depressive symptoms, anxiety symptoms, and academic stress each partially mediate the relationship between childhood maltreatment and suicide risk. The persistence of significant direct effects after including the mediators indicates that psychological distress explains an important, but not exclusive, portion of this association, a pattern of partial mediation consistent with contemporary developmental models proposing that suicidal behavior emerges through multiple interacting pathways rather than a single causal mechanism (Turecki et al., 2019; Turecki and Brent, 2016). This mirrors findings in comparable university-student samples, where childhood trauma effects on suicidal ideation were only partially attenuated after accounting for psychological mediators such as alexithymia and resilience (Gao et al., 2025), although other mediation studies using different mediators (depression, psychache, self-harm) have reported full attenuation, underscoring that the degree of mediation observed is sensitive to which mediators are modeled (Halim et al., 2025).

Within this pattern, depression emerged as the mediator most strongly affected by childhood maltreatment, consistent with extensive evidence identifying depression as the principal proximal predictor of suicidal ideation across both clinical and community populations, and specifically as the single strongest correlate of suicidal ideation and attempt among medical students in meta-analytic pooled estimates (Nelson et al., 2017; Ribeiro et al., 2018; Seo et al., 2021). Childhood maltreatment has repeatedly been associated with earlier onset, greater severity, and increased recurrence of depressive disorders, a pattern corroborated by causal-inference research (Zhang et al., 2025), while depression itself contributes to suicidal thinking through hopelessness, negative cognitive schemas, anhedonia, and diminished future orientation. Anxiety symptoms also emerged as a significant, partly independent mediator: in adolescents and young adults with first depressive episodes, anxiety severity has been shown to mediate the maltreatment–suicide attempt relationship independently of depression, accounting for roughly a quarter of the total effect (Chen et al., 2021), and among medical undergraduates specifically, elevated generalized anxiety has been directly correlated with prior trauma exposure (Vagiri et al., 2025).

By contrast, academic stress represented the mediator most closely and proximally tied to suicide risk itself in the present models. Unlike childhood maltreatment, which reflects a distal developmental exposure, academic stress constitutes an immediate contextual challenge experienced throughout medical training, and the present findings suggest it may function as the environmental context through which latent developmental vulnerabilities become clinically expressed: students with histories of childhood maltreatment may perceive academic demands as more overwhelming because early adversity alters emotional appraisal, coping strategies, and stress responsiveness. This interpretation aligns with the stress sensitization hypothesis, which proposes that early adversity lowers the threshold at which subsequent stressors elicit psychological distress (McLaughlin et al., 2010) and is consistent with broader university-student mediation research showing that academic stress exerts much of its effect on suicidal ideation through depression (Castañeda Quirama et al., 2025; Tyagi et al., 2026).

Depression, anxiety, and academic stress should not be interpreted as independent mediators acting in isolation, but as interconnected dimensions of psychological distress that mutually reinforce one another; chronic academic stress may precipitate depressive symptoms, anxiety may intensify stress perception, and depression may reduce coping resources, creating a self-perpetuating cycle that progressively increases suicide risk. This is consistent with evidence that academic burnout, a construct closely related to chronic academic stress, influences suicidal ideation indirectly through repetitive negative thinking rather than through a significant direct pathway, further supporting the layered, sequential nature of these mechanisms (Pourmohammad et al., 2026).

The finding that childhood maltreatment retained a significant direct association with suicide risk after accounting for psychological distress suggests that additional mechanisms not evaluated here (emotional dysregulation, hopelessness, self-compassion, resilience, distress tolerance, attachment insecurity, and social support) also contribute to suicidal vulnerability (Angelakis et al., 2020; Yao et al., 2023). Notably, self-compassion has been shown to moderate the depression-mediated pathway itself, with the indirect effect of childhood trauma on suicide risk through depression emerging as significant only at moderate-to-high levels of self-compassion (Song et al., 2026). The present mediation models should therefore be interpreted as identifying clinically meaningful pathways rather than a complete explanation.

From a clinical perspective, because childhood maltreatment cannot be modified retrospectively while depressive symptoms, anxiety, and academic stress are dynamic and treatable, these results support a shift from risk identification based exclusively on childhood adversity toward trauma-informed prevention strategies targeting current psychological functioning—systematic mental health screening, early treatment of depressive and anxiety symptoms, stress-management programs, and institutional efforts to reduce excessive academic burden.

Taken together, these findings support a theoretically plausible statistical pathway in which childhood maltreatment is associated with psychological distress, which in turn is associated with suicide risk. Among the mediators examined, depressive symptoms showed the strongest association with childhood maltreatment, whereas academic stress was most closely associated with suicide risk. This pattern positions psychological distress as a clinically relevant bridge between developmental vulnerability and suicidal risk, while avoiding assumptions about temporal or causal directionality. A natural next step would be to examine which institutional, interpersonal, or dispositional factors are associated with academic stress in this population, given its close relationship to suicide risk and its potential role within the pathways linking early adversity to suicidal outcomes.

The consistent, dominant role of depressive symptoms as a mediator across both maltreatment subtypes is compatible with neurobiological and stress-sensitization frameworks proposing that childhood adversity recalibrates the threshold at which later stressors elicit emotional and physiological distress (McLaughlin et al., 2010), and with evidence that childhood maltreatment is associated with persistent HPA-axis dysregulation and altered frontolimbic functioning (Fujisawa et al., 2018; Heim et al., 2008; Teicher et al., 2016). This converges with research showing that general stress appraisal and perceived stress mediate the pathway from childhood trauma to depression, anxiety, and other suicide-related constructs such as defeat and entrapment, with perceived stress showing particularly robust mediation of the trauma–depression link, supporting stress appraisal as a plausible upstream mechanism feeding into the more specific academic-stress pathway examined in the present study (Wang et al., 2025). Although the present study did not include biological measures, this framework offers a coherent explanation for why individuals with histories of childhood adversity may experience heightened emotional reactivity when confronted with the demands of medical training. Emotional maltreatment, in particular, has historically received less attention than physical or sexual abuse despite evidence that it may exert equally, or even more, persistent effects on psychological functioning, interfering with the development of secure attachment, self-worth, and adaptive emotion regulation during critical developmental periods (Teicher et al., 2016).

Importantly, these findings should not be interpreted as suggesting that childhood adversity inevitably leads to suicidal behavior. Most individuals exposed to childhood maltreatment never develop suicidal ideation, indicating that developmental vulnerability is probabilistic rather than deterministic; protective factors such as supportive relationships, adaptive coping, resilience, and timely mental health intervention may substantially modify these trajectories (Turecki et al., 2019).

### 4.2 Strengths and limitations

This study's strengths include a relatively large sample of second-year medical students, the use of validated and widely used psychometric instruments, a multidimensional conceptualization of psychological distress examining depressive symptoms, anxiety, and academic stress as parallel mediators, and, to our knowledge, being among the first studies in a large Latin American medical student sample to evaluate this developmental pathway, addressing a gap in a literature dominated by East Asian and Western populations (Halim et al., 2025; Nan et al., 2023).

Several limitations should be acknowledged. The cross-sectional design precludes establishing temporal or causal relationships; although the proposed model is theoretically grounded and supported by longitudinal evidence elsewhere, including trajectory studies showing that maltreatment predicts persistently elevated suicidal ideation over time (Nan et al., 2023), directionality cannot be confirmed here, and prospective or causal-inference designs (e.g., Mendelian randomization) are needed (Zhang et al., 2025). All variables were self-reported, which may introduce recall bias, social desirability, or underreporting, particularly for sensitive experiences; structured clinical interviews or multimodal measures would strengthen future work. Participants were recruited from a single medical school in Mexico, which may limit generalizability to other institutions or cultural contexts. In addition, participants were drawn from a single academic year (second-year students), and approximately 30% of the eligible population ultimately provided complete data; although this proportion is consistent with classroom-based survey studies, we cannot rule out selection bias if non-participating or excluded students differed systematically in psychological distress or maltreatment history from those included, and findings may not generalize to students at other stages of medical training (e.g., clinical or internship years). The observed partial mediation indicates that mechanisms beyond depression, anxiety, and academic stress (emotion regulation, hopelessness, self-compassion, which has been shown to moderate the depression-mediated pathway) (Song et al., 2026) remain unexplored and merit inclusion in future models. Finally, because this study focused on emotional and physical maltreatment, other forms of childhood adversity (including sexual abuse, which can retain a direct effect on suicide risk independent of the mechanisms that explain other maltreatment subtypes) (Huang and Hou, 2023) were not examined separately and warrant broader trauma-informed designs in future research.

## 5 Conclusion

Despite these limitations, the present findings provide robust evidence supporting a developmental model of suicide risk among medical students, in which childhood maltreatment contributes to psychological vulnerability while depressive symptoms, anxiety, and academic stress function as clinically meaningful and potentially modifiable pathways linking early adversity with suicide risk. These results also support the growing movement toward trauma-informed medical education: understanding students' developmental histories, alongside routine screening for current symptoms, may help identify students who are particularly vulnerable to academic stress before severe psychological symptoms emerge, and incorporating trauma-informed principles (psychologically safe learning environments, faculty training, early mental health screening, and interventions targeting emotional regulation and stress management) may complement conventional suicide-prevention strategies (Whitaker et al., 2024). Medical school does not create vulnerability; rather, it may reveal and amplify developmental vulnerabilities established long before students enter medical training. Suicide risk therefore reflects the interaction between early-life adversity and the psychological demands of medical education, highlighting psychological distress as a modifiable pathway for trauma-informed prevention.

## Data Availability

Data supporting the findings of this study may be made available by the authors upon reasonable request. Access to individual-level data is subject to applicable ethical requirements and considerations regarding participant privacy. Inquiries regarding data availability should be directed to the corresponding author.
